# Isoimperatorin Induces Apoptosis of Nasopharyngeal Carcinoma Cells via the MAPK/ERK1/2 Signaling Pathway

**DOI:** 10.1155/2020/2138186

**Published:** 2020-03-09

**Authors:** Jie Liu, Lan He, Jing Hu, Kairui Li, Fangliang Zhou, Mei Hu, Jingjing Luo, Lan Song, Yingchun He

**Affiliations:** ^1^Graduate School, Hunan University of Chinese Medicine, Changsha, Hunan 410208, China; ^2^Medicine School, Hunan University of Chinese, Changsha, Hunan 410208, China; ^3^Hunan Provincial Key Laboratory for the Prevention and Treatment of Ophthalmology and Otolaryngology Diseases with Traditional Chinese Medicine, Changsha, Hunan 410208, China; ^4^Hunan Provincial Ophthalmology and Otolaryngology Diseases Prevention and Treatment with Traditional Chinese Medicine and Visual Function Protection Engineering and Technological Research Center, Changsha, Hunan 410208, China

## Abstract

**Objective:**

To investigate the effect of isoimperatorin on nasopharyngeal carcinoma CNE2 cell apoptosis and the role of the MAPK/ERK1/2 signaling pathway in inducing apoptosis.

**Methods:**

Real-time cellular analysis technology (RTCA) and MTT were used to detect cell proliferation; Annexin V-FITC/PI dual-fluorescence flow cytometry analysis, Hoechst 33342 staining, and mitochondrial membrane potential detection kit were used to detect cell apoptosis; western blot was used to detect protein expression.

**Results:**

Different concentrations of isoimperatorin (10 *μ*M, 20 *μ*M, 20 *μ*M, 20 *μ*M, 20 *μ*M, 20 *μ*M, 20 *μ*M, 20

**Conclusion:**

Isoimperatorin can induce nasopharyngeal carcinoma CNE2 cell apoptosis through the MAPK/ERK1/2 signaling pathway.

## 1. Introduction

Nasopharyngeal carcinoma (NPC) is among the most common malignant cancer of the head and neck in southern China. Tumors originate from the top and lateral wall of the nasopharyngeal cavity and are primarily treated using radiotherapy and chemotherapy. Due to the significant toxicity and side effects associated with these treatment methods, it is urgent to find alternative methods that are safer and more effective. The imbalance of proliferation and apoptosis in tumor cells is an important factor for its unrestricted proliferation and is regulated by cell signaling pathways. Our team found that the MAPK/ERK1/2 signaling pathway is highly activated in nasopharyngeal carcinoma cells, and inhibition of this pathway can induce apoptosis in nasopharyngeal carcinoma cells. Therefore, studying the MPAK/ERK1/2 signaling pathway may provide targets for treating nasopharyngeal cancer.

Traditional Chinese medicines and their monomers have gradually become a hot spot in the research field due to their extensive functions, immune modulatory function, and efficacy in treating drug-resistant conditions. Isoimperatorin (ISOIMP) studied in this article is a naturally occurring coumarin class compound and is one of the active ingredients in *Changium smyrnioides* Wolff, *Angelicae dahuricae* Radix, *Notopterygium incisum*, and *Glehnia littoralis*. It has been found in the previous studies that isoimperatorin has a wide range of pharmacological effects as follows: can promote the differentiation of adipocytes; may prevent diabetes [1]; inhibits the activity of melanocytes; promotes the expression of melanin transport related protein Rab27a; reduces the melanocyte melanin content [2, 3]; reduces the expression of nitric oxide and tumor necrosis factor-*α* (TNF-*α*) in fibroblast synovial cells [4]; improves the mitochondrial function; protects the acute liver injury induced by carbon tetrachloride [5]; relaxes the blood vessels [6]; and increases the breast cancer cell radiotherapy sensitivity [7]. Besides, isoimperatorin has anti-inflammatory, analgesic, and antitumor effects and can induce apoptosis as well as inhibit the proliferation of gastric cancer cells [8–10]. To date, no reports have examined whether isoimperatorin has an antitumor effect on nasopharyngeal cancer. Thus, the aim of this study was to test the effects of different isoimperatorin concentrations on the proliferation and apoptosis of nasopharyngeal carcinoma CNE2 cells and to explore its mechanism of action.

## 2. Materials

### 2.1. Cell Line

Human nasopharyngeal carcinoma CNE2 cells were purchased from Beijing North Natron Biotechnology Research Institute and subcultured in our laboratory.

### 2.2. Drugs

Isoimperatorin was purchased from Shanghai Jinsui Bio-Technology Co., Ltd and cisplatin (CIS) from Qilu Pharmaceutical Co., Ltd., National Pharmaceutical Standard H20023460.

### 2.3. Reagents

RPMI-1640 medium (Hyclone), fetal bovine serum (Gibco), DMSO (Amresco), MTT (Biosharp), Annexin V-FITC/PI apoptosis kit (MULTI SCIENCES), *β*-actin Mouse mAb, Survivin Rabbit mAb, XIAP Rabbit mAb, Phospho-c-Raf Rabbit mAb, Phospho-MEK Rabbit mAb, Phospho-p44/42 MAPK (ERK1/2) Rabbit mAb (Cell Signaling TECHNOLOGY), Rabbit Anti-PCNA Polyclonal Antibody, Rabbit Anti-Bax polyclonal antibody, and Rabbit Anti-Bcl-2 Polyclonal Antibody (Bioss) were used.

### 2.4. Instruments

Double single-sided purification workbench (SW-CJ-2FD) (Suzhou Purification Equipment Co., Ltd), CO_2_ incubator Heraeus HERAcell 150i (Thermo Fisher Scientific), automatic enzyme label analyzer (ELX800) (BioTek), dual-fluorescence flow cytometry cellometer image cytometer (K2) (Nexcelom), Cytation™ 5 cell imaging multifunctional detection system (BioTek), Odyssey-CLX two-color infrared fluorescence imaging system (Gene), and real-time cellular analysis technology (RTCA xCElligence, ACEA Biosciences Inc) were used.

## 3. Methods

### 3.1. Cell Culture

Nasopharyngeal carcinoma CNE2 cells were cultured in RPMI-1640 medium supplemented with 10% fetal bovine serum in an incubator at 37°C and 5% CO_2_. Cells were passaged every 3 days, and cells in the logarithmic growth phase were used for experiments.

### 3.2. RTCA Monitoring of Cell Proliferation in Real Time

Cells in the logarithmic growth phase were seeded into a real-time label-free cellular analysis technology (RTCA) specific culture plate at 3000 cells per well. Cells were grouped as follows: control (solvent control group), ISOIMP (10 *μ*M, 20 *μ*M, 30 *μ*M, and 40 *μ*M) group [1], and cisplatin-positive control (CIS, 4 *μ*g/mL) [11].

### 3.3. MTT Assay for Cell Proliferation

Cells in the logarithmic growth phase were seeded in 96-well plates at 3000 cells per well and incubated overnight. Next, the media was removed and replaced with 200 *μ*L of medium containing different concentrations of the drug. A total of 5 replicate wells were set for each concentration. After 24, 48, and 72 hours, the drug containing media was discarded, and 100 *μ*L of 10% MTT solution was added to each well and incubated for 4 hours. The MTT was then discarded, and 100 *μ*L of DMSO was added to each well. After shaking for 10 minutes in the dark, a microplate reader was used to measure the absorbance (*A*) at 490 nm. Cell viability was analyzed using the following formula: cell viability of the drug group = (drug group *A* value − blank group *A* value)/(control group *A* value − blank group *A* value) *∗* 100%.

### 3.4. Apoptosis Detection by AnnexinV-FITC/PI Double Fluorescent Staining

The cells were incubated with three different concentrations (10 *μ*M, 20 *μ*M, and 40 *μ*M) of isoimperatorin for 48 h. The cells were then digested with trypsin without EDTA, collected in EP tubes, centrifuged at 1000 rpm for 3 minutes, and washed twice with PBS. Next, 50 *μ*L of 1 × binding buffer was added and cells were resuspended. To stain the cells, 5 *μ*L of FITC to each tube and 5 *μ*L of PI stain were added and incubated for 15 min. To stop the reaction, 50 *μ*L of 1 × binding buffer were added. Then, the apoptosis rate was quantified, and the results were analyzed.

### 3.5. Observing Apoptosis by Hoechst 33342 Staining

After 48 hours of treatment with different drug concentrations, the culture solution was discarded, cells were washed twice with PBS, stained with 10 *μ*g/mL Hoechst 33342 staining solution for 30 min, and washed twice with PBS. Cells were then covered with PBS and photographed in the Cytation™ 5.

### 3.6. Mitochondrial Membrane Potential Detection Kit for Detecting Changes in Cell Mitochondrial Membrane Potential

After being treated with different concentrations of drugs for 48 h, the culture solution was discarded, washed twice with PBS, stained with 1 *μ*g/mL JC1 staining solution for 30 min, and washed twice with PBS. Cells were then covered with PBS and photographed in the Cytation™ 5.

### 3.7. Detection of Protein Expression Levels by Western Blot

After 48 h of drug treatment, the culture dish was placed on ice, washed twice with PBS, and treated with 50 *μ*L of RIPA Lysis Buffer for 30 min. The protein was collected and quantified using a BCA protein quantification kit, followed by electrophoresis on 12% SDS-PAGE gel. The protein was then transferred to a PVDF membrane and blocked using 5% skim milk for 1 h. The membrane was cut into the relevant pieces and incubated with primary antibody at 4°C overnight. The membrane was washed 5 times with TBST for 5 minutes each time and then incubated with secondary antibody for 1.5 hours at room temperature in the dark. The membrane was washed 5 times in TBST for 5 min each time. Finally, the membrane was scanned using an Odyssey-CLX two-color infrared fluorescence imaging system, and the band signal value was analyzed in Image Studio Ver 5.2 software. The expression level of each protein was normalized to *β*-actin.

## 4. Statistical Analysis

SPSS22.0 statistical software was used for data processing. The experimental measurements followed a normal distribution, expressed by ± *s*, single factor design, and measurement data for multiple groups were compared using one-way ANOVA to satisfy the homogeneity of variance. Multiple comparisons were performed using LSD, and the variance was not tested by the Dunnet T3 test. The rank-sum test was used for data not compliant with the normal distribution. *P* < 0.05 was considered statistically significant.

## 5. Results

### 5.1. Isoimperatorin Inhibits CNE2 Cell Proliferation

**Figure 1:**
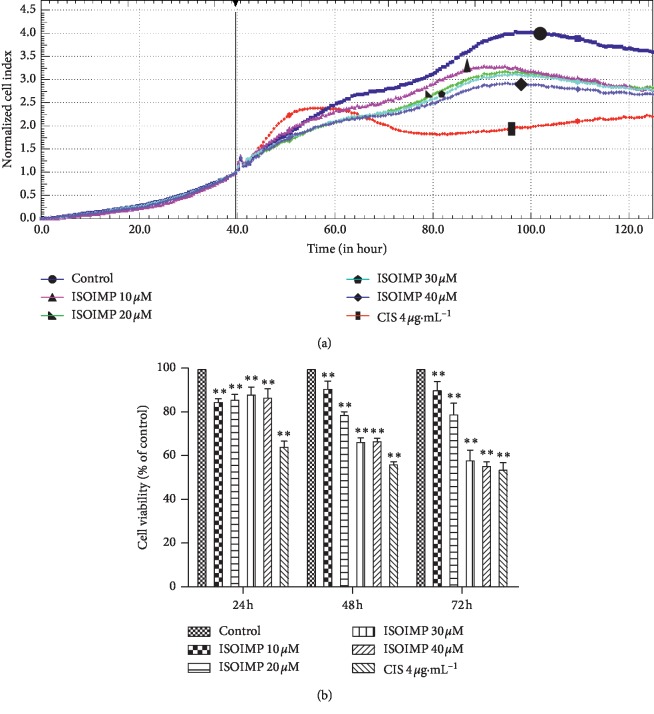
(a) RTCA analysis of the inhibitory effect of isoimperatorin on CNE2 cell proliferation at different concentrations (10 *μ*M, 20 *μ*M, 30 *μ*M, and 40 *μ*M). (b) MTT analysis of the inhibitory effects of different concentrations (10 *μ*M, 20 *μ*M, 30 *μ*M, and 40 *μ*M) of isoimperatorin on CNE2 cell proliferation at 24 h, 48 h, and 72 h vs control group: ^*∗∗*^*P* < 0.01.

CNE2 cells were continuously monitored by RTCA for at least 72 hours to observe the effects of isoimperatorin treatment on proliferation. The results showed that all tested concentrations of isoimperatorin could inhibit the proliferation of nasopharyngeal carcinoma CNE2 cells after 24 hours of treatment (Figure 1(a)). MTT showed (Figure 1(b)) that, compared with the solvent control group, all tested concentrations of isoimperatorin significantly inhibited cell proliferation after 24 h, 48 h, and 72 h of treatment. The inhibitory effect was most obvious after 48 h treatment (*P* < 0.01) and acted in a concentration-dependent manner, with the 30 *μ*M and 40 *μ*M drug groups showing significantly stronger inhibition than the 10 *μ*M or 20 *μ*M drug group.

### 5.2. Isoimperatorin Induces Apoptosis in CNE2 Cells

**Figure 2:**
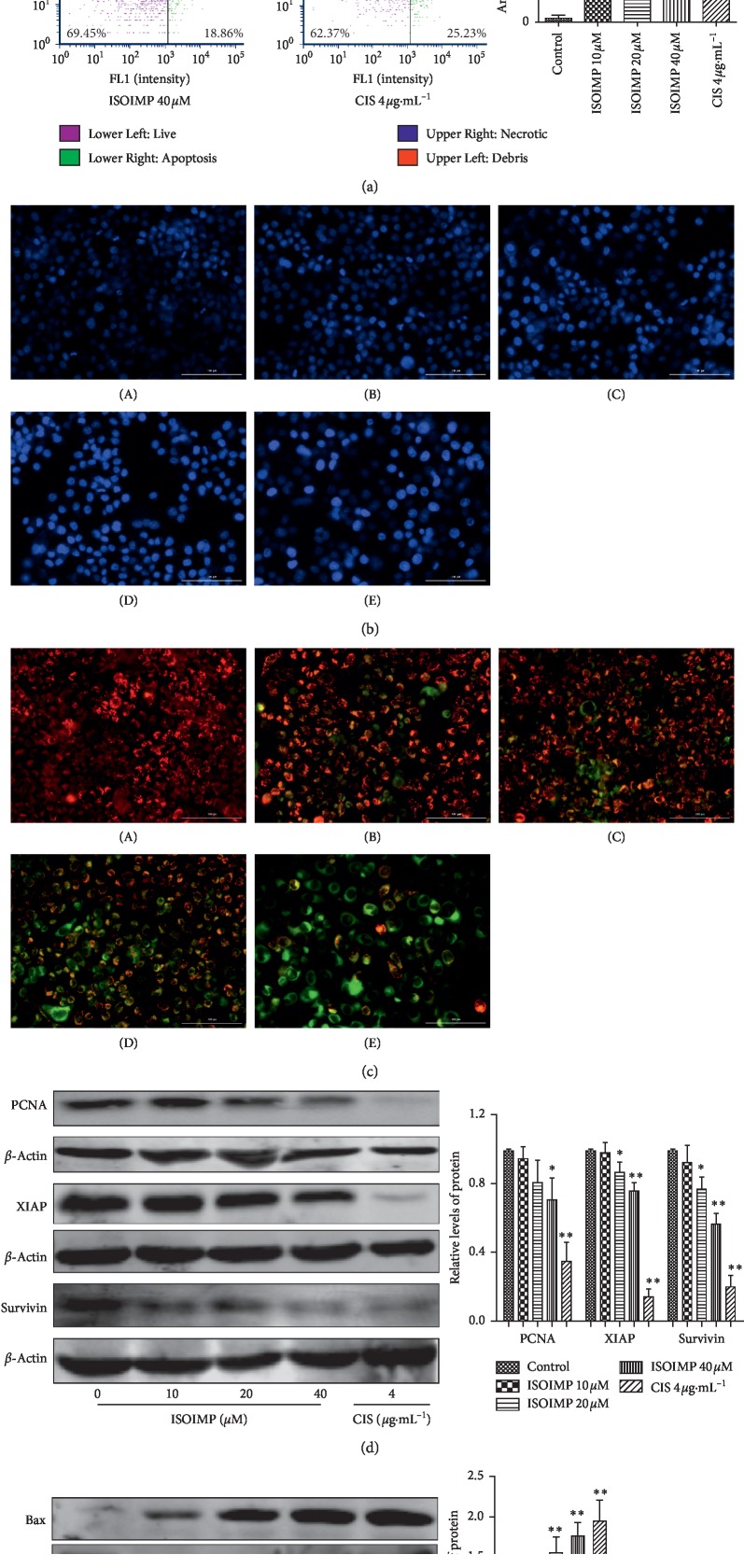
(a) Dual-fluorescence flow cytometry cellometer image cytometer (K2) was used to detect the change of apoptotic rate of CNE2 cells after isoimperatorin intervention. The apoptotic rate in the 10 *μ*M group was 23.06 ± 2.00%, the 20 *μ*M group was 22.90 ± 3.47%, and the 40 *μ*M group was 29.06 ± 1.25% vs control group: ^*∗∗*^*P* < 0.01. Cytation™ 5 cell imaging multifunctional detection system detects changes in the nucleus (b) and cell membrane potentials (c) of the cells after the intervention of isoimperatorin. (A) Control; (B)ISOIMP 10 *μ*M; (C) ISOIMP20 *μ*M; (D)ISOIMP 40 *μ*M; (E) CIS 4.0 *μ*g·mL^–1^(bar=100 *μ*m, 200x). The nucleus appears bright blue with nuclear condensation. The increase in green fluorescence indicates a decrease in CNE2 cell membrane potentials and cell apoptosis. Isoimperatorin inhibits the expression of proliferation-related and apoptosis-related proteins in CNE2 cells. Western blot analysis of the effects of isoimperatorin at different concentrations on the relative expression of PCNA, XIAP, and survivin (d) and Bax and Bcl-2 (e) in CNE2 cells vs control group: ^*∗*^*P* < 0.05; ^*∗∗*^*P* < 0.01.

The results of Annexin V-FITC/PI double fluorescent staining (Figure 2(a)) showed that, after 48 h of treatment with 10 *μ*M, 20 *μ*M, and 40 *μ*M isoimperatorin, it significantly induced the apoptosis of nasopharyngeal carcinoma cells as compared with the solvent control group (*P* < 0.01). Hoechst 33342 staining (Figure 2(b)) showed normal nuclei which appear light blue and have a full and uniform morphology. After 48 hours of isoimperatorin treatment, the nuclei are stained bright blue and show apoptotic features including nuclear pyknosis. The mitochondrial membrane potential detection kit method showed (Figure 2(c)) that, compared with the control group, the green fluorescence of the drug group was more and more, indicating that isoimperatorin can reduce the mitochondrial membrane potential of nasopharyngeal carcinoma cells and cause early apoptosis of cells. Protein expression levels were analyzed after 48 h of drug treatment. Compared with the solvent control group, the expression levels of the proliferation-related protein PCNA and the antiapoptosis proteins XIAP, survivin (Figure 2(d)), and Bcl-2 (Figure 2(e)) in the 20 *μ*M and 40 *μ*M isoimperatorin groups significantly decreased, while expression of the proapoptotic protein Bax increased (Figure 2(e)).

### 5.3. Effect of Isoimperatorin on the Expression of Key Proteins in the MAPK/ERK1/2 Signaling Pathway

**Figure 3:**
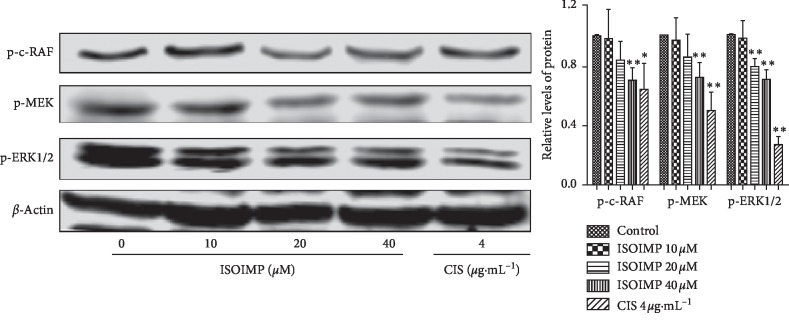
Isoimperatorin inhibits phosphorylation of the MAPK/ERK1/2 signaling pathway. Western blot analysis of the expression of p-c-RAF, p-MEK, and p-ERK1/2 in CNE2 cells. vs control group: ^*∗*^*P* < 0.05; ^*∗∗*^*P* < 0.01.

Expression levels were measured after 48 h of drug treatment by western blot. Compared with the solvent control group, the expression levels of key proteins p-c-RAF, p-MEK, and p-ERK1/2 in the MAPK/ERK1/2 signaling pathway were significantly decreased following the treatment with each concentration of isoimperatorin. The difference was statistically significant (*P* < 0.05) (Figure 3).

### 5.4. Role of the MAPK/ERK1/2 Signaling Pathway in Isoimperatorin-Induced CNE2 Cell Apoptosis

**Figure 4:**
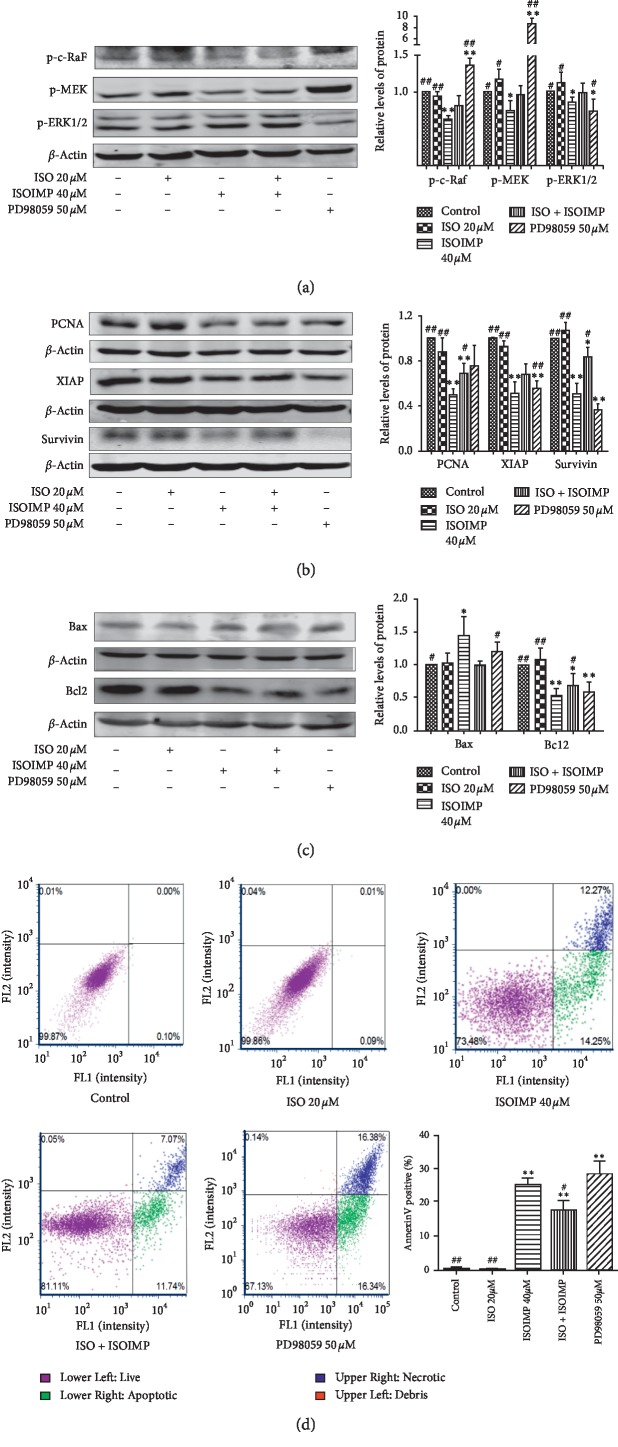
Effect of isoimperatorin on CNE2 cell apoptosis is attenuated by ISO. Western blot analysis shows the expression of p-c-RAF, p-MEK, and p-ERK1/2 (a), PCNA, XIAP, and survivin (b), and Bax and Bcl-2 (c) in CNE2 cells. vs control group: ^*∗∗*^*P* < 0.01; vs ISOIMP group, ^#^*P* < 0.05; ^##^*P* < 0.01; dual-fluorescence flow cytometry cellometer image cytometer (K2) was used to detect the change of apoptotic rate of CNE2 cells after ISO and isoimperatorin intervention (d) vs control group: ^*∗∗*^*P* < 0.01; vs ISOIMP group, ^#^*P* < 0.05; ^##^*P* < 0.01.

CNE2 cells were treated with a MAPK/ERK1/2 signaling pathway activator termed ISO either as a single treatment in the ISO group or in combination with isoimperatorin in the ISO combination group to further clarify whether isoimperatorin induces CNE2 cell apoptosis by inhibiting the MAPK/ERK1/2 signaling pathway. Activation of the MAPK/ERK1/2 signaling pathway via ISO significantly reduced the efficacy of isoimperatorin-mediated downregulation of key signaling pathway proteins p-c-RAF, p-MEK, and p-ERK1/2 (Figure 4(a)), proliferation-related protein PCNA, and antiapoptosis proteins XIAP, survivin (Figure 4(b)), and Bcl-2 (Figure 4(c)), significantly reducing its efficacy in upregulating the proapoptotic protein Bax (Figure 4(c)). Flow cytometry results further confirmed that isoimperatorin-induced nasopharyngeal carcinoma cell apoptosis was significantly reduced after activation of the MAPK/ERK1/2 signaling pathway by ISO compared with the isoimperatorin group alone (*P* < 0.01) (Figure 4(d)).

## 6. Discussion

Nasopharyngeal carcinoma occurs in an insidious location, and the operation required to treat it is difficult. In China, chemoradiotherapy combined with traditional Chinese medicine is the most commonly used treatment and leads to a noticeable improvement in the patient survival rate [12–14]. However, the clinical treatment of nasopharyngeal carcinoma still faces major obstacles which must be overcome, including the serious side effects of radiotherapy and chemotherapy, drug resistance, recurrence, and metastasis. In recent years, molecular -targeted therapy for malignant tumors has become a popular treatment method. In this treatment strategy, drugs are selected to directly affect the target cells and change their biological behavior at the molecular level including proliferation, apoptosis, metastasis, autophagy, and pyroptosis, but to have no effect on normal cells. As natural medicines, traditional Chinese medicines have the advantages of a high level of safety, specific curative effect, and ability to avoid developing drug resistant disease. Researchers have been studying the efficacy and mechanism of traditional Chinese medicines, such as berberine [15], baicalein [16, 17], arctiin [18], nitidine chloride [19], and other monomers [20] and have found them to exert obvious anticancer effects. However, significant future research will be needed on related fields in order to better develop the use of traditional Chinese medicines.

The previous studies have shown that isoimperatorin effectively inhibits the proliferation of gastric cancer and induces its apoptosis [8–10]. Wang Meng [21] also confirmed that isoimperatorin has obvious antitumor activity. However, in-depth research into its potential effects on related forms of cancer has not been carried out. In the present study, we confirmed by RTCA and MTT that different concentrations of isoimperatorin had significant inhibitory effects on nasopharyngeal carcinoma CNE2 cell proliferation and that the inhibitory effect increased as the concentration increased. Prior studies as well as the RTCA results presented here suggest that isoimperatorin may cause DNA damage resulting in loss of the cell's ability to undergo replication and transcription [22, 23]. Flow cytometry analysis showed that, compared with the solvent group, isoimperatorin induced the CNE2 cell apoptosis. The drug remained active for 48 hours and primarily caused early cellular apoptosis. The cells were also observed by Hoechst 33342 staining at the same timepoint. The nuclei within the solvent group were dyed a uniform light blue color, while the isoimperatorin group nuclei showed blue fluorescence with different intensities and a condensed nuclear morphology which was most apparent in the high concentration group. Apoptotic bodies were observed in cells in the late stages of apoptosis. Proliferating cell nuclear antigen (PCNA) is closely related to cellular DNA synthesis and can be used to label cells that are mitotically active; interestingly, it is highly expressed in nasopharyngeal carcinoma cells. X-related apoptosis inhibitory protein (XIAP) is the strongest inhibitor of apoptosis in the IAP family [24–26], and survivin is also an apoptosis-inhibiting protein widely expressed in tumor cells [19]; together, these factors promote DNA replication and can influence the proliferation and apoptosis of tumor cells. The proapoptosis gene Bax and the antiapoptosis gene Bcl-2 belong to the Bcl-2 family and play opposing roles in apoptosis regulation. Studies have confirmed that a shift in the Bax/Bcl-2 ratio is an important factor contributing to the low apoptosis rate of tumor cells and that this ratio is targeted by various antitumor drugs [27]. Our western blot results showed that isoimperatorin can inhibit the PCNA, XIAP, and survivin expression, upregulate the Bax expression, downregulate the Bcl-2 expression, and increase the Bax/Bcl-2 ratio, indicating that isoimperatorin-mediated inhibition of CNE2 cell proliferation is closely related to inhibition of DNA synthesis and induction of apoptosis-related protein expression.

Mitogen-activated protein kinase (MAPK) regulates many important cellular physiological functions, and the ERK pathway is primarily involved in the regulation of cell proliferation, apoptosis, and differentiation. Under normal circumstances, a variety of growth factor receptors mediate signal transduction through the activation of ERK, thus enabling cell proliferation. However, in tumor cells, abnormal activation of the ERK signaling pathway can promote malignant transformation and proliferation [28–34]. Prior studies have shown that the MAPK/ERK signaling pathway is closely related to the occurrence and development of nasopharyngeal carcinoma [28], and the Chinese herbal compound Yiqi Jiedu Decoction inhibits nasopharyngeal carcinoma cell proliferation by inhibiting this pathway [11]. In this study, the expression levels of the key MAPK/ERK1/2 signaling pathway proteins p-c-RAF, p-MEK, and p-ERK1/2 were measured. They found that isoimperatorin significantly inhibited the activation of the MAPK/ERK1/2 signaling pathway. In the present work, activation of the MAPK/ERK1/2 signaling pathway in nasopharyngeal carcinoma cells by a specific activator reduced the ability of isoimperatorin to downregulate the key proteins p-c-RAF, p-MEK, and p-ERK1/2. At the same time, the effect of isoimperatorin on nasopharyngeal carcinoma cells apoptosis was also reduced, that is, the MAPK/ERK activator dampened the ability of isoimperatorin to induce apoptosis, indicating that isoimperatorin may prevent nasopharyngeal carcinoma proliferation and induce apoptosis via the MAPK/ERK1/2 signaling pathway. At the same time, we found that PD98059 upregulated the expression level of p-MEK, which may be a negative feedback regulation mechanism, which inhibited p-MEK, causing a large number of upstream proteins to activate, thereby promoting the phosphorylation of MEK [35].

## Data Availability

The data used to support the findings of this study are available from the corresponding author upon request.
